# An Effective Technique for Endoscopic Resection of Advanced Stage Angiofibroma

**Published:** 2014-01

**Authors:** Mojtaba Mohammadi Ardehali, Seyyed Hadi Samimi, Mehdi Bakhshaee

**Affiliations:** 1 *Otorhinolaryngology Research Center, Amir Alam Educational Hospital, Tehran University of Medical Sciences, Tehran, Iran.*; 2 *Sinus and Surgical Endoscopic Research Center, School of Medicine, Mashhad University of Medical Sciences, Mashhad, Iran.*

**Keywords:** Angiofibroma, Complication, Endoscopy, Nasal, Novel technique, Outcome, Recurrence, Tampon, Technique

## Abstract

**Introduction:**

In recent years, the surgical management of angiofibroma has been greatly influenced by the use of endoscopic techniques. However, large tumors that extend into difficult anatomic sites present major challenges for management by either endoscopy or an open-surgery approach which needs new technique for the complete en block resection.

**Materials and Methods:**

In a prospective observational study we developed an endoscopic transnasal technique for the resection of angiofibroma via pushing and pulling the mass with 1/100000 soaked adrenalin tampons. Thirty two patients were treated using this endoscopic technique over 7 years. The mean follow-up period was 36 months. The main outcomes measured were tumor staging, average blood loss, complications, length of hospitalization, and residual and/or recurrence rate of the tumor.

**Results:**

According to the Radkowski staging, 23,5, and 4 patients were at stage IIC, IIIA, and IIIB, respectively. Twenty five patients were operated on exclusively via transnasal endoscopy while 7 patients were managed using endoscopy-assisted open-surgery techniques. Mean blood loss in patients was 1261± 893 cc. The recurrence rate was 21.88% (7 cases) at two years following surgery. Mean hospitalization time was 3.56 ± 0.6 days.

**Conclusion:**

Using this effective technique, endoscopic removal of more highly advanced angiofibroma is possible. Better visualization, less intraoperative blood loss, lower rates of complication and recurrence, and shorter hospitalization time are some of the advantages.

## Introduction

Juvenile nasopharyngeal angiofibroma (JNA) is a rare benign vascular tumor originating from the sphenopalatine foramen and found exclusively in males, mostly during adolescence. Many treatment modalities have been used for tumor management; among the approaches only surgery and radiotherapy have proven to be effective (1–3). Surgery is the treatment of choice for JNA and for many years several different open-surgery approaches have been used to manage this tumor with acceptable morbidity and mortality. Traditional surgical approaches including transpalatal, lateral rhinotomy and midfacial degloving were used successfully for a long time; however, these approaches are accompanied by various cosmetic and functional co-morbidities (4).

In recent years, the use of endoscopic techniques has greatly influenced the management of small to moderate JNA tumors; however, the role of these techniques in treating larger tumors is controversial but they may play some role as an adjunct to standard open techniques (5). 

Even when the tumor is embolized, the vascular entity of the tumor and a bloody field during surgery make it difficult to excise the tissue completely, especially when the tumor extends to less accessible anatomic areas. In this study we developed a new technique using tampons soaked in a solution of adrenaline to compress the tumor and dissect it by using pushing force in a bloodless field. This approach can lead to the complete removal of advanced tumors under good visual guidance.

## Materials and Methods

We have developed a transnasal endoscopic technique for the resection of angiofibroma. Thirty two patients were treated using this technique over 7 years (1999–2008). The mean follow-up period was 36 months (range 18–95 months). Tumor staging, average blood loss, complications, length of hospitalization, residual, and/or recurrence rate of tumor were the main outcomes measured.

***Surgical technique***

**Fig 1:**
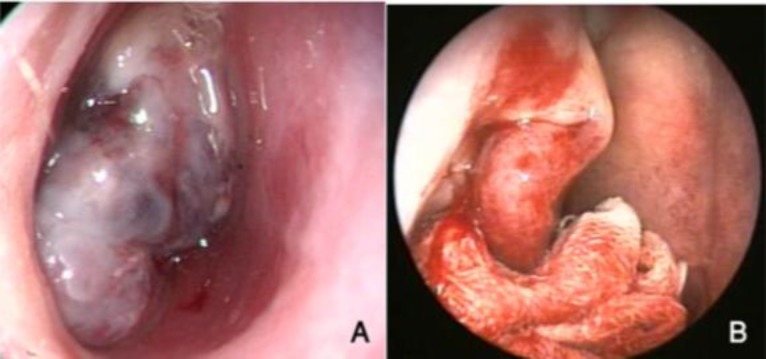
Endoscopic view of JNA before use of the tampon (A) and after pushing the tumor to the posterior by applying pressure with the tampon (B).

In a 3 cases embolization was performed before the surgical procedure. In the remaining cases no preparatory procedures were used prior to surgery. The endoscopic procedure was performed as follows: Under general anesthesia, the nose was packed with pledgets soaked in a vasoconstrictor (adrenalin 1/100000) and the tampon positi-

oned in such a way that it pushed the tumor towards the posterior (nasopharynx) (Fig. 1). 

This maneuver let the surgeon work in a bloodless field and be able to gradually expose the nasal anatomical structures step by step anteriorly. Transnasal endoscopic instrumentation was applied by using 0°, 30°, and occasionally 70° telescopes. 

**Fig 2:**
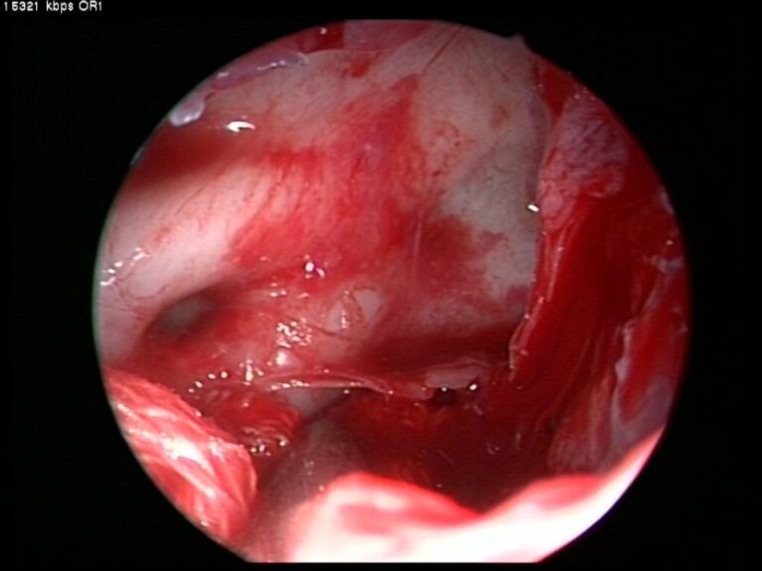
Endoscopic view of the sphenoid sinus while the tumor is pushed inferiorly by tampon in a bloodless field

In most cases, we firstly dissected and separated the mass in its most anterior attachement. As JNA insertion is subperiosteal the entire tumor is usually subperiosteal and submucosal and an incision was made through the nasal mucosa at a site close to the JNA and force applied to dissect the JNA from this point. The tumor was then progressively detached by posteromedially pushing and pulling downward, so that the tumor was pushed to the nasopharynx. For better visualization uncinectomy, wide antrostomy, ethmoidectomy, sphenoidotomy, and partial middle turbinectomy were performed according to the extension of the mass. However, in many cases the tumor mass and its extension gave us enough options to follow without the need to remove any specific anatomical area. Tumor removal at its boundaries in the sphenoid and maxillary sinus was usually completed by progressive traction and detachment (Fig. 2).

**Fig 3:**
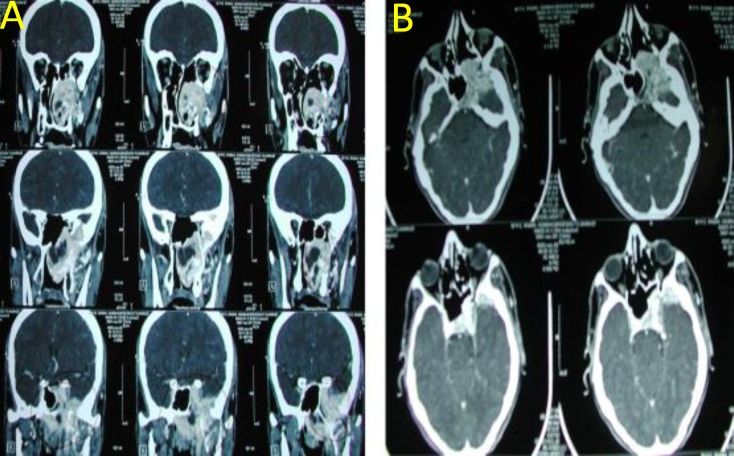
Preoperative coronal (A) and axial (B) views of contrast CT scan of a patient with advanced angiofibroma who was referred for surgery due to nasal obstruction and epistaxis

**Fig 4:**
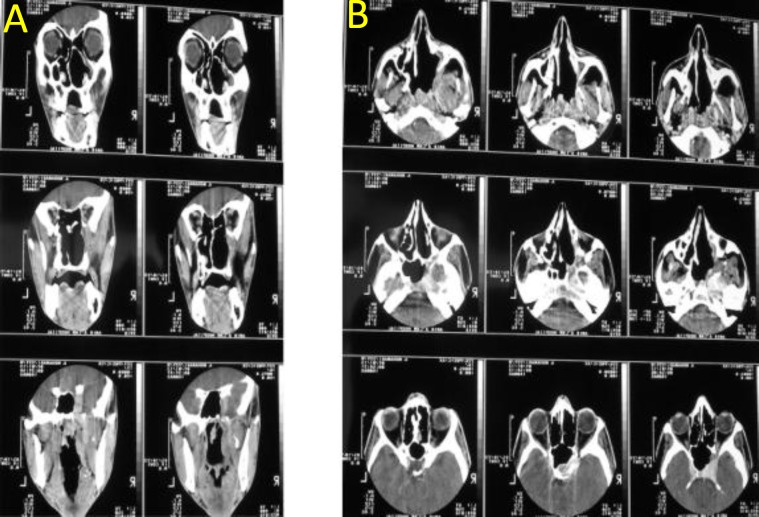
Postoperative coronal (A) and axial (B) views of contrast CT scan of the patient six months after tumor removal via exclusively endoscopic transnasal surgery

Following this step the region of the sphenopalatine foramen was exposed by resecting the posterior half of the middle turbinate, as well as performing an antrostomy and possibly a posterior ethmoidectomy, which led to exposure of the orbital surface. Krison’s punch was then used to remove the posterior wall of the maxillary sinus. This was often an easy procedure due to thinning of the bone caused by the tumor. Thus, virtual removal of the entire posterior wall of the sinus was possible. After releasing the tumor from its boundaries and pushing it down into the nasopharynx and oropharynx, en bloc resection of the tumor was possible by transoral extraction. The operative field was then carefully inspected to detect any possible remnants. Extensive drilling of the basisphenoid and clival area where the tumor digitations are probable was performed in the last step. Additional homeostasis was carried out as necessary. In all cases there was no need to pack the nasal fossa. In tumors with cranial base destruction or that extended medially and under the cavernous sinus, the optic nerve and carotid artery were identified using endoscopy and the tumor resected from these structures under direct visual guidance (Fig. 2). This resection could be done in most primary cases as the tumor did not invade these soft tissue structures, but rather pushed them away. In the case of secondary surgeries the story is totally different as fibrosis and tumor adhesion to these vital structures could increase the risk of morbidity as the surgeon tries to dissect the tumor. The patients received follow-up monitoring at 1-month intervals for 6 months, and then every 3 months up to 2 years following surgery and then once a year thereafter (Fig. 3,4). 

Complete nasal and nasopharyngeal endoscopy was performed during these visits. A CT scan with contrast and/or MRI with contrast were obtained at 3 months post-surgery and at the end of year 1 then once a year thereafter. Residual tumor sizes were recorded and compared with other visits. This study was approved by ethic committee of Tehran University of Medical Sciences and all the patient were informed completely about study and signed informed consents.

## Results

Thirty two patients with a mean age of 16.19 ± 5.3 years (range 7–37 years) were enrolled in the study. Nasal obstruction (93.75. %), epistaxis (62.5%), and facial swelling (18.75%) were the most common symptoms. According to the Radkowski staging, 23, 5, and 4 patients were at stage IIC, IIIA, and IIIB, respectively. Twenty five patients were operated on exclusively via transnasal endoscopy while 7 patients were managed with endoscopy-assisted open-surgery techniques. These 7 cases were in stage IIC in six cases and only one was in stage IIIA. The vast extension of the tumor laterally into the infratemporal fossa; made it necessary that to tumor is released via ipsilateral gingivobuccal incision and pushed medially for en bloc resection. Embolization was performed only in 3 (9.4. %) cases. Mean blood loss in patients was 1261±893cc. The recurrence rate of the tumor was 21.9% (7 cases). Mean hospitalization was 3.56 ± 2.9 days. Complications included 2 patients with cheek paresthesia, 2 with epiphora, 1 patient who had transient weakness of cranial nerves II, III and VI, and 2 cases of cavernous sinus injury. Erosion of the nostril due to the pressure and friction related to instrument usage occurred in some patients but resolved without any scars in all cases (Table 1).

**Table 1:** Outcome measures among 32 advanced angiofibroma cases

**Case No**	**Age** **(Year)**	**Stage**	**Approach**	**Embolization**	**Blood Loss (cc)**	**Hospitalization** **(day)**	**Recurrence**
1	12	IIC	Double	No	320	2	Yes
2	17	IIIA	Double	No	2100	4	No
3	18	IIIA	Endoscopic	No	500	2	No
4	20	IIIB	Endoscopic	No	600	3	No
5	11	IIIB	Endoscopic	Yes	1500	7	Yes
6	19	IIC	Double	No	1600	3	No
7	17	IIIB	Endoscopic	No	850	2	No
8	10	IIIA	Endoscopic	No	1500	3	No
9	14	IIC	Endoscopic	No	3000	1	No
10	13	IIC	Endoscopic	Yes	1000	2	No
11	19	IIIA	Endoscopic	No	4500	17	Yes
12	14	IIC	Double	No	1000	3	No
13	14	IIC	Endoscopic	Yes	1000	2	No
14	16	IIC	Endoscopic	No	1000	2	No
15	21	IIC	Endoscopic	No	300	3	No
16	10	IIC	Double	No	750	3	No
17	12	IIC	Endoscopic	No	300	4	No
18	18	IIC	Endoscopic	No	1250	2	Yes
19	15	IIC	Endoscopic	No	1000	3	No
20	24	IIC	Endoscopic	No	1000	2	No
21	21	IIC	Endoscopic	No	4500	2	No
22	37	IIIB	Endoscopic	No	8500	8	No
23	17	IIIA	Endoscopic	No	2500	2	Yes
24	14	IIC	Double	No	2500	5	No
25	15	IIC	Endoscopic	No	1000	5	No
26	7	IIC	Endoscopic	No	900	5	No
27	15	IIC	Endoscopic	No	1050	3	No
28	15	IIC	Double	No	1250	4	No
29	18	IIC	Endoscopic	No	2000	2	No
30	14	IIC	Endoscopic	No	300	4	Yes
31	12	IIC	Endoscopic	No	1500	3	No
32	19	IIC	Endoscopic	No	1000	1	Yes

## Discussion

Today, parallel to the evolution of endoscopic instrumentation and techniques, endoscopic approaches have increasingly become more popular. Many studies have reported endoscopic removal as the approach of choice for small to medium JNAs (6-10). This approach if meticulously carried out is very effective, resulting in less disability in patients, decreases in the duration of hospitalization, and lower rates of intraoperative bleeding. Other advantages of the endoscopic approach include avoidance of surgical scars on the face, resection of the least amount of normal soft tissue, and avoidance of the destruction of facial bones and occurrence of late facial deformity (5,9).

Although the use of endoscopy in the resection of small JNA is supported by acceptable results in a number of studies that have been published in recent years, large JNA continues to present an important surgical challenge, both via traditional open approaches or endoscopic ones 9. During the recent years an increase in the number of exclusive endoscopic approaches has been reported in the literature (6,11-15). However, the conclusions and evidence surrounding surgical resection of large JNA remains mixed. For example, none of the 7 cases of Wormald and Van Hasselt, which were resected endoscopically, had a disease stage greater than Radowski’s IIC (13). In a series by Nicolai et al. 4 of the 15 patients were classed IIIA or IIIB and only 1 patient presented a residual lesion after 24 months (12). The conclusion of the series stated that advanced lesions were better treated by external approaches. Roger et al., in a series of 20 cases from which 9 were Radowski’s types IIIA, favored a wholly endoscopic approach and reported a complete excision in 7 cases and small asymptomatic remnants in 2 cases (6). Combining endoscopy with open surgery is another possibility that has also been considered in the medical records 16-18. The choice between an exclusively endoscopic approach and surgery facilitated by endoscopy depends on the localization of the tumor as well as the experience of the surgeon. For example, Onerci et al. emphasized that in high-stage tumors, a surgeon must be prepared to shift to a combined approach, due to possible presence of extensive residual tumor (14). 

Our study contained 32 advanced cases (stage IIC and above) of which 25 were resected via the exclusive endoscopic approach and 7 cases were managed using an endoscope-assisted approach. Three cases had a wide extension to the lateral wall of the infratemporal fossa so we easily released them from the surrounding tissues by using buccolabial incisions and pushed the mass into the nasal cavity. Mair et al. and Khalifa et al. have also reported using such a technique to bring the tumor into endoscopy view and access in cases with lateral extension (15,16). 

The greatest indicator of success in any tumor surgery is the rate of recurrence. Recurrence is commonly seen in JNA and ranges from 25% to 46% (17,19-21). We had 21.9 % recurrence after two years of follow-up which is less than that previously reported by Gullane et al.; however, a usual rate of relapse of 25% for stage IIC and 40% to 50% for stage III tumors has been reported in other studies (17,19,20). Refinement of the operation technique by our method and addressing the pseudopods extension of the tumor into the petrygoid root and sphenoid body (tumor growth in a digitations pattern into the basisphenoid and clivus which was managed by extensive drilling) and also the soked tampone in adrenalin solution which eliminate the bleeding not only from the injured mucosal area near the tumor by pharmacologic effect but also from the tumor itself due indirect compression on the mass during surgery may explain the low rate of recurrence in our study. The vascular nature of this tumor is an obstacle for endoscopic surgery, so any procedure that can decrease the amount of bleeding, such as hypotension, embolization, meticulous surgical planning, and technique refinement enables a surgeon to be more successful regardless of the approach. However, Mohammadi et al. did not show that embolization plays a significant role in decreasing bleeding during operations on JNA. In our technique at all stages the tampon soaked in adrenalin solution helped us to compress the mass, put pressure indirectly on the tumor in a bloodless field, and finally resect the tumor22. By these measures we were able to control bleeding and complete the operation in all cases, even in advanced situations.

## Conclusion

The endoscopic approach that we have developed is an effective technique that allows removal of even large-sized juvenile angiofibroma safely with a low morbidity. Improvements in preoperative assessment, refinement of the operation technique, and instrumentation make the endoscopic removal of most juvenile nasopharyngeal angiofibroma possible. 
